# Burst spinal cord stimulation for central neuropathic pain: Two case reports

**DOI:** 10.1097/MD.0000000000024628

**Published:** 2021-02-12

**Authors:** Lim-joon Yoon, Deok-yeong Kim

**Affiliations:** Department of Neurosurgery, Nowon Eulji Medical Center, Eulji University, Seoul, Korea.

**Keywords:** analgesia, central nervous system diseases, neuralgia, spinal cord stimulation

## Abstract

**Introduction:**

Central neuropathic pain can result from any type of injury to the central nervous system. Treatment of central neuropathic pain is very challenging. Recently, a novel stimulation paradigm, called burst stimulation, has been presented as an excellent alternative in a group of patients with intractable central neuropathic pain. We report 2 cases where burst spinal cord stimulation (SCS) was applied in patients with neuropathic pain due to spinal cord injury (SCI) or traumatic brain injury.

**Patient concerns:**

A 52-year-old man who underwent posterolateral fusion surgery for a T12 bursting fracture after a fall 11 years prior developed disabling pain in the anterolateral part of his right thigh. His neuropathic pain following SCI was refractory to various treatment modalities. A 65-year-old man had complained of intractable, cold, throbbing, and shooting pain mainly in his left lower limb during rehabilitation since undergoing a craniotomy 9 years prior for multiple brain injuries caused by a motorcycle accident.

**Diagnosis:**

Both of these 2 cases were diagnosed with central neuropathic pain syndrome caused by SCI or traumatic brain injury.

**Interventions:**

Burst SCS were proposed to alleviate the significant refractory pains that were resistant to various medications and stimulation was delivered to the patient in an alternating pattern between traditional tonic and burst waveforms.

**Conclusion:**

The efficacy of burst SCS in central neuropathic pain is desirable considering the severity of pain in such patients, the refractory nature of their pain, and the paucity of alternative therapeutic options.

## 1 Introduction

Central neuropathic pain can result from any type of injury to the central nervous system (CNS), such as stroke, spinal cord injury, or multiple sclerosis.^[1]^ While the mechanism responsible for peripheral neuropathic pain is quite known, it is much less understood about the mechanism underlying central neuropathic pain. For this reason, central neuropathic pain is very challenging to treat and has little reaction to pharmacological agents routinely used for peripheral neuropathic pain.^[2]^ Conventional spinal cord stimulation (SCS) providing tonic stimulation is considered ineffective for central neuropathic pain despite the fact it has been well established as a safe and effective treatment of pain arising from a wide variety of etiologies.^[3,4]^ Recent technological advancements have allowed new SCS waveforms to be used for successful SCS treatment. Burst SCS(b-SCS), which was designed by De Ridder et al in 2010^[5]^ to improve the efficacy and expanded the applicability of SCS, has shown good patient outcomes for more than 20 studies including randomized controlled trial (RCT). However, the efficacy of b-SCS for central neuropathic pain has not been adequately explored, and there are only a few reports of its use in a small number of patients.^[6]^ Here, we describe 2 case studies of patients with refractory neuropathic pain due to central nervous system (CNS) lesions who were successfully treated with b-SCS, as well as a review of the literature on the effect of b-SCS.

## 2 Case report

### 2.1 Case 1

**Figure 1:**
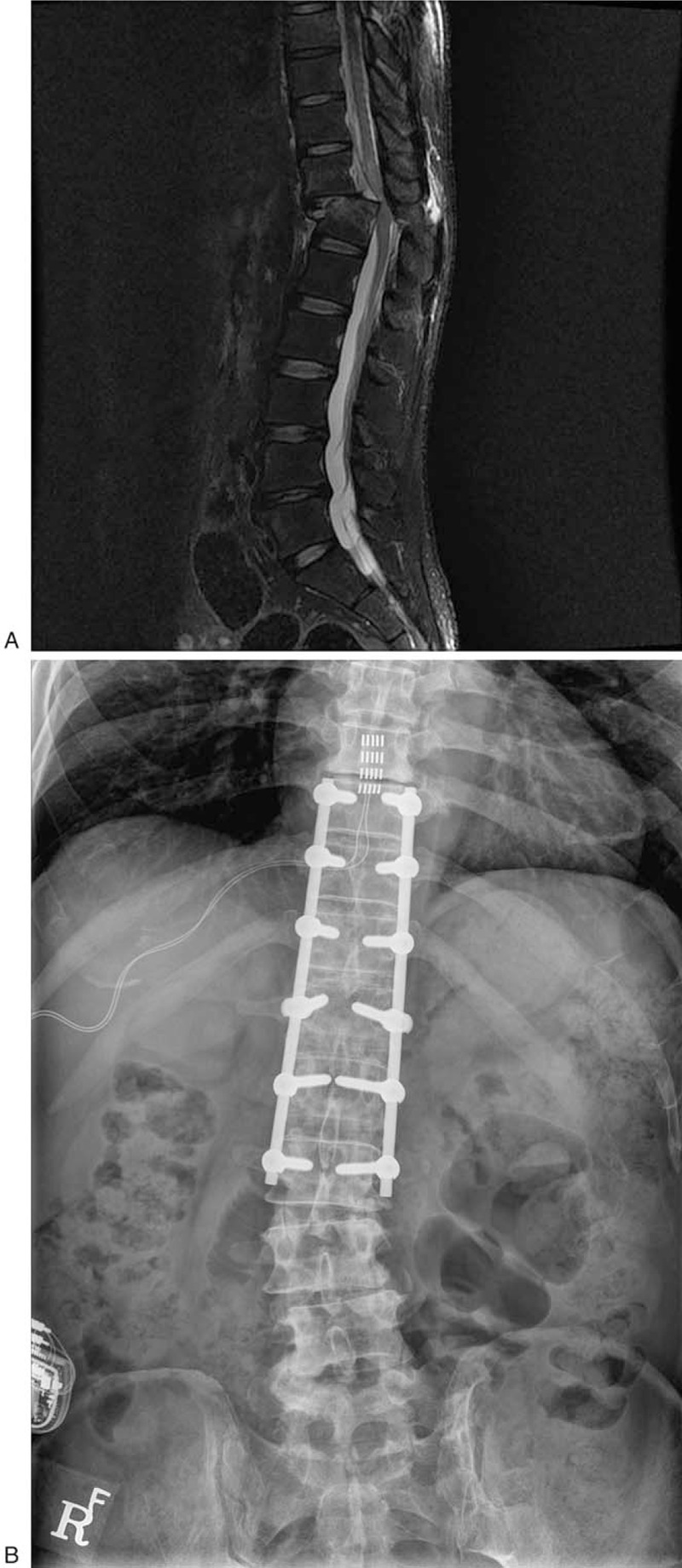
MR and pain film images obtained in Case 1. (A) Preoperative MRI showing a T12 bursting fracture combined with dislocation; (B) Plain x-ray showing an SCS electrode placed at the T9 level and a permanent rechargeable neurostimulation system.

A 52-year-old man who underwent posterolateral fusion surgery for a T12 bursting fracture combined with dislocation diagnosis after a fall 11 years ago was referred to the neurosurgical department with intractable neuropathic pain in the anterior of his right thigh. At that time, even after surgery, he had the posttraumatic sequalae, such as paraplegia, anesthesia, and neurogenic bladder and bowels. The extent of his previous spinal cord injury (SCI) corresponded to American Spinal Injury Association scale A.^[7]^ Immediately after the onset of sensorimotor dysfunction of the lower limb, the disabling pain developed in the anterolateral part of his right thigh. Pain intensity on visual analog scale was recorded at over 8/10 (0 meaning no pain, 10 meaning the worst imaginable pain), and the characteristics of pain were noted to be stabbing, pinprick, and squeezing sensations which occurred paroxysmally, with about 5 attacks lasting 3 to 4 minutes each per hour. Neuropathic pain in SCI can be classified as at-level or below-level, according to its location with regards to the level of the lesion.^[8]^ This patient was diagnosed with at-level SCI neuropathic pain having a T12 neurological level of injury because the pain was localized to the right anterolateral thigh, which corresponds to the L2 dermatome. His neuropathic pain following his SCI was refractory despite various treatment modalities, including pharmacological, interventional, and psychological approaches. After intensive discussion at a multidisciplinary meeting, our team decided to try b-SCS in an attempt to alleviate this patient's pain. A trial of tonic SCS was excluded from the discussion because neurophysiological testing showed absent cortical somatosensory evoked potentials (SEP) in the affected area, while SEP of the median nerve showed normal cortical responses. A 5-column Penta paddle lead (Abbott, Plano, TX) was surgically placed at the T9 level, and burst stimulation with an intraburst 500 Hz frequency, overall 40 Hz frequency, and 1 ms pulse width was attempted (Fig. 1). After confirming that the pain intensity and frequency were significantly reduced, permanent rechargeable neurostimulation system (Prodigy, Abbott, Plano, TX) insertion followed. On the 1-year follow-up mark, the patient reported undiminished efficacy of the stimulation, which had reduced the frequency and intensity of his pain after surgery by half. The patient has provided informed consent for the publication of this case report and accompanying images.

### 2.2 Case 2

**Figure 2:**
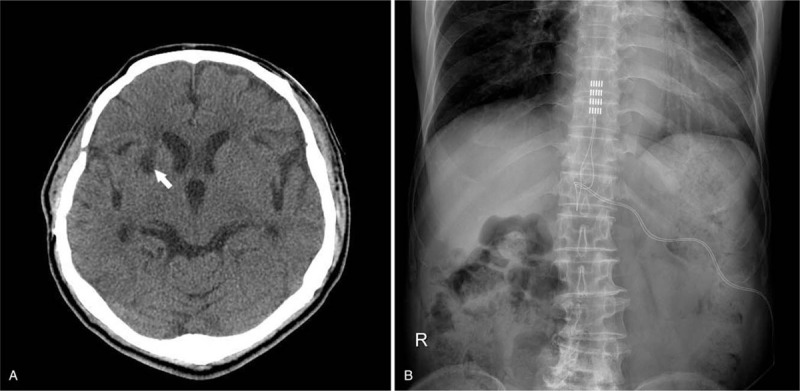
CT and pain film images obtained in Case 2. (A) Axial CT scan shows a right focal hypodense lesion in the basal ganglia (arrow). (B) Plain x-ray shows a paddle electrode placed at the T9 level and an implantable pulse generator.

The second case report describes a 65-year-old man with intractable, cold, throbbing, and shooting pain mainly in his left lower limb. He had complained of this neuropathic pain during rehabilitation since undergoing a craniotomy 9 years ago for multiple brain injuries caused by a motorcycle accident. Several back surgeries had been performed over the past few years to correct the cause of this neuropathic pain after it was misdiagnosed as being caused by lumbar spinal lesions. However, after these spinal operations, the patient remained in pain. He was then referred to the neurosurgical department with intractable neuropathic pain. The patient was assessed by a pain specialist, and eventually was diagnosed with central neuropathic pain syndrome caused by the previous traumatic damage done to the sensory pathways of the central nervous system. Testing for SEP revealed prolonged latency in the left tibial nerve above the T12 level. Based on several published reports suggesting that SCS may effectively treat central neuropathic pain syndromes, this modality was proposed to alleviate his significant refractory pain that was resistant to various medications. To maximize pain-control effects, a 5-column Penta lead to the T9 level and a rechargeable neurostimulation system (Prodigy, Abbott, Plano, TX) were surgically placed (Fig. 2), and stimulation was delivered to the patient in an alternating pattern between traditional tonic and burst waveform. For tonic stimulation, the pulse width was programmed in the range of 100 to 500 ms, with frequencies typically between 30 and 100 Hz, and at amplitudes producing comfortable paresthesia according to the patient's perception. Through the first 6 months of stimulation, the patient's pain relief was maintained at about two-thirds his prestimulation levels. However, his pain gradually returned to its prestimulation state, and about 3 years after the initial surgery, both stimuli were again used alternatingly, which resulted in about a one-third improvement in the patient's pain. The patient has provided informed consent for the publication of this case report and accompanying images.

## 3 Discussion

**Figure 3:**
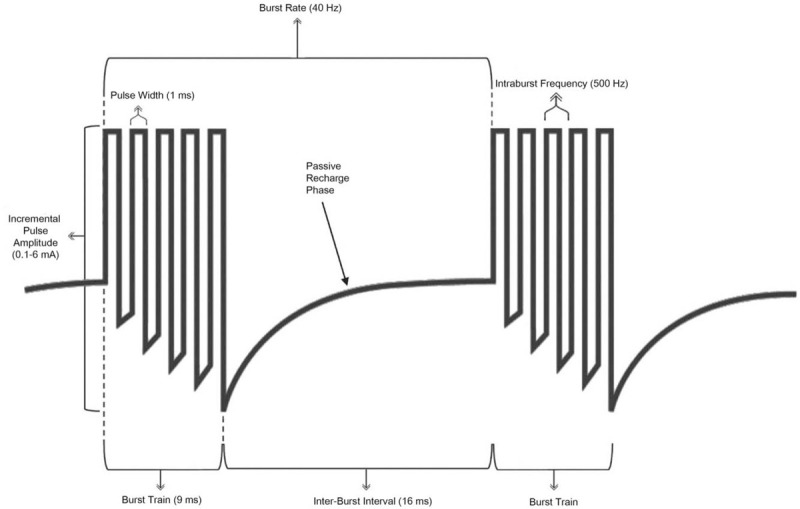
Illustration of burst waveforms with passive recharge.

SCS offers a treatment option that has minimal side effects and that is relatively safe and potentially reversible. Since the U.S. Food and Drug Administration (FDA) first approved traditional tonic SCS (t-SCS) in 1989 to relieve chronic pain from nerve damage,^[9]^ t-SCS has been commonly used for the treatment of chronic intractable pain of the trunk and limbs, including unilateral or bilateral pain associated with failed back surgery syndrome, intractable low back and leg pain, complex regional pain syndrome types I and II, and neuropathic pain.^[10,11]^ Despite clinical successes of t-SCS in treating a subset of chronic neuropathic pain syndromes,^[12]^ several limitations have prevented its growth to a generalized treatment. First, although most patients are able to cope with paresthesia, a significant proportion report that the sensation is unpleasant, particularly with positional changes.^[13]^ Second, not all patients treated with t-SCS experience sufficient pain relief. In general, the reported success rate for t-SCS is roughly 50% pain relief in approximately 50% to 70% of patients.^[14]^ Third, the effectiveness of t-SCS seems to decrease when used over the course of years, as shown in various long-term follow-up studies.^[15]^ b-SCS, first described by De Ridder,^[5]^ delivers packets of 40-Hz bursts with 5 spikes at 500-Hz spike frequency, a pulse width of 1 ms, an inter-burst interval of 1 ms, and inter-burst interval followed by a passive recharge phase between burst train. (Fig. 3) Burst firing is more similar to normal nerve activity and need less temporal integration to activate cortical neurons.^[16]^ The charge per burst has shown to be important for the inhibition of pain transmission neurons.^[17]^ More importantly, since it requires a lower amplitude than t-SCS, b-SCS is typically paresthesia-free when administered properly.^[5]^ Many recent studies have been steadily carried out in an attempt to understand the basic neurophysiologic effects of b-SCS. Data from several human sources, such as electrophysiological and imaging studies, have suggested that modulation of fibers in the human nervous system with b-SCS seems to have different underlying mechanisms than those underlying the modulation seen with t-SCS; specifically, it has been suggested that b-SCS modulates the affective emotional system to a larger extent than t-SCS, while also modulating the sensory-discriminative system to a similar extent.^[18]^ These characteristics of b-SCS are believed to contribute significantly to alleviating the fear and anxiety caused by the evolution of acute to chronic pain.^[19]^ Research is still underway on how b-SCS works in the human nervous system, and our hope is that additional data will continue to elucidate these mechanisms in the near future.

**Figure 4:**
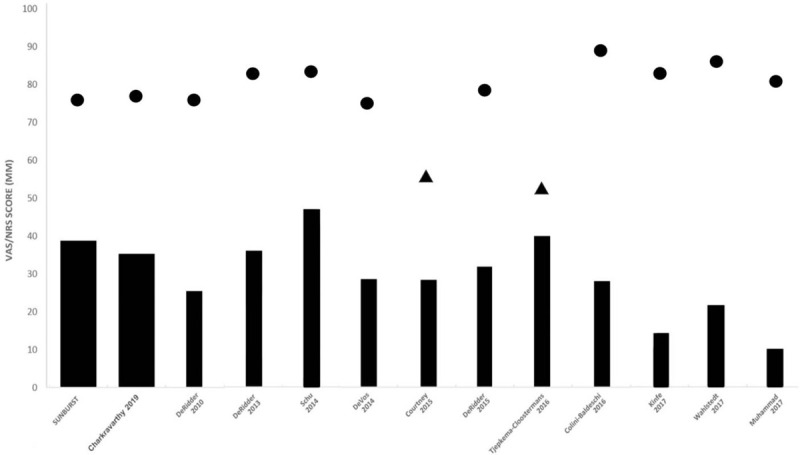
Pain scores (visual analog scale or numeric rating scale) from previous studies. Points represent pain scores at baseline before the operation. Triangles represent pain scores reported during tonic stimulation. Bar heights represent final pain scores with burst stimulation.

Since the first trial of b-SCS for pain neuromodulation was reported in 2010, several studies evaluating burst stimulation have demonstrated its efficacy at relieving pain, while also improving functional and psychological outcomes.^[20–22]^ Among these, the study designed to investigate the clinical effects of b-SCS with the most objective and reliable methods can be cited as the multicenter, randomized, unblinded, crossover Success Using Neuromodulation with BURST (SUNBURST) study published by Deer et al in 2018.^[23]^ A main result of this study established the noninferiority of improvement in pain intensity after 3 months of burst stimulation compared to 3 months of tonic stimulation (*P* < .001). In addition to demonstrating noninferiority, the superiority of burst stimulation over tonic stimulation was also demonstrated (*P* < .017). Another important result was that more than two-thirds (70.8%) of subjects preferred burst stimulation, even though all subjects responded to tonic stimulation during the trial evaluation. Imaging data from a sub-study of the SUNBURST study using fluorodeoxyglucose positron emission tomography showed that b-SCS modulates the dorsal anterior cingulate cortex involved in the medial pain pathway, leading to enhanced processing of the affective and motivational control of pain compared to tonic stimulation.^[24]^ These results suggest that the additional and significant impact of b-SCS on the medial pathways, which represent the affective emotional system, resulted in the profound patient preference for b-SCS even though the somatosensory measures of pain relief were similar between the b-SCS and tonic SCS groups. In 2019, Chakravarthy et al^[6]^ performed a meta-analysis, which included SUNBURST data, to provide a more realistic reflection of outcomes with b-SCS and to avoid potential unforeseen biases seen with smaller sample sizes. This meta-analysis reviewed fifteen articles with a combined sample size of 427 cases within a publication date range of 2010 to 2019 (Fig. 4). All included studies used technology manufactured by St. Jude/Abbott, delivering the BurstDR^TM^ waveform which is the only FDA-approved burst stimulation. Each article's level of evidence was rated according to a standard methodology, resulting in 1 study being identified as level 1 evidence, 6 as level 2, and the rest as level 3 or level 4. The pooled analysis showed that the average pooled pain score was 49.2 with t-SCS and 36.7 with b-SCS, a 12.5-point difference. The clinical importance of this benefit was supported by the large majority of subjects who preferred b-SCS over t-SCS. A notable finding in the pooled analysis of pain intensities was that the difference between burst vs tonic stimulation (12.5 points) was more pronounced than the difference reported in the SUNBURST RCT (5.2 points). In addition to the preferential effect of b-SCS on pain intensity, patient-reported outcomes, such as questionnaires about mood, disability, or quality of life, were consistently improved with b-SCS compared with t-SCS. These improvements in patient-reported outcomes indicate that b-SCS may provide benefits not only for pain intensity, but also for the holistic pain experience. Given these results, this review is of great value in that it provides quantitative evidence of the overall effectiveness of b-SCS for the first time through the pooled analyses with specific objective data.

## 4 Conclusion

This paper has provided 2 cases where b-SCS was applied in patients with neuropathic pain due to SCI and traumatic brain injury, as well as a comprehensive summary of previous studies on b-SCS. Although the mechanism of b-SCS is not fully understood, the effectiveness of analgesia and the psychometric benefits on central neuropathic pain shown by b-SCS is important given the severity of pain in these patients, the refractory nature of their pain, and the paucity of alterative therapeutic options. A further prospective, controlled study with a larger population of patients is needed to provide stronger evidence of the efficacy of b-SCS and to define the patient populations that are most likely to benefit from burst stimulation, as well as tonic stimulation.

## Author contributions

**Conceptualization:** Deok ryeong Kim.

**Data curation:** Lim joon Yoon.

**Formal analysis:** Lim joon Yoon.

**Investigation:** Lim joon Yoon.

**Resources:** Deok ryeong Kim.

**Supervision:** Deok ryeong Kim.

**Writing – original draft:** Lim joon Yoon, Deok ryeong Kim.

**Writing – review & editing:** Deok ryeong Kim, Lim-joon Yoon.

## Abbreviations

Abbreviations: b-SCS = burst spinal cord stimulation, CNS = central nervous system, FDA = Food and Drug Administration, RCT = randomized controlled trial, SCI = spinal cord injury, SCS = spinal cord stimulation, SEP = somatosensory evoked potentials, SUNBURST = Success Using Neuromodulation with BURST, t-SCS = tonic spinal cord stimulation.
